# Tamoxifen is not effective in good prognosis patients with hepatocellular carcinoma

**DOI:** 10.1186/1471-2407-6-196

**Published:** 2006-07-24

**Authors:** Ciro Gallo, Ermelinda De Maio, Massimo Di Maio, Giuseppe Signoriello, Bruno Daniele, Sandro Pignata, Annalisa Annunziata, Francesco Perrone

**Affiliations:** 1 Department of Medicine and Public Health, Second University, Naples, Italy; 2 Clinical Trials Unit, National Cancer Institute, Naples, Italy; 3 G. Rummo Hospital, Benevento, Italy; 4 Medical Oncology B, National Cancer Institute, Naples, Italy

## Abstract

**Background:**

Large randomised clinical trials and systematic reviews substantiate that tamoxifen is ineffective in improving survival of patients with hepatocellular carcinoma (HCC). However, a recent report suggested that the drug might prolong survival among patients with well preserved liver function. The aim of this paper is to validate this hypothesis.

**Methods:**

We used the updated database of the phase 3 randomised CLIP-1 trial that compared tamoxifen with supportive therapy. Primary endpoint was overall survival. Treatment arms were compared within strata defined according to the Okuda stage and the CLIP-score. Survival differences were tested by the Log-rank test.

**Results:**

Tamoxifen was not effective in prolonging survival in Okuda I-II subgroup (p = 0.501). Median survival times were equal to 16.8 (95%CI 12.7–18.5) months for tamoxifen and 16.8 (95%CI 13.5–22.4) months for the control arms; 1-year survival probabilities were equal to 58.8% (95%CI 51.7–65.8) and 59.4 (95%CI 52.5–66.2), respectively. Similar results were observed in the better CLIP subgroup (score 0/1), without evidence of difference between the two treatment arms (p = 0.734). Median survival times were equal to 29.2 (95%CI 20.1–36.4) months with tamoxifen and 29.0 (95%CI 23.3–35.2) months without; 1-year survival probabilities were equal to 80.9% (95%CI 72.5–89.3) with tamoxifen and 77.1% (95%CI 68.6–85.7) for the control arm.

**Conclusion:**

The recent suggestion that tamoxifen might be effective in the subgroup of patients with better prognosis is not supported by a reanalysis of the CLIP-1 trial. Tamoxifen should no longer be considered for the treatment of HCC patients and future trials of medical treatment should concentrate on different drugs.

## Background

At the end of '90, large randomised clinical trials showed the ineffectiveness of tamoxifen in the treatment of hepatocellular carcinoma (HCC) both in advanced [1,2] and potentially curable patients [3]. Since then many reviews repeatedly asserted there was no evidence of any effect of tamoxifen in improving survival of HCC patients [4-9].

Recently, the lack of a survival advantage was further confirmed by a French trial with 420 HCC patients randomly assigned tamoxifen or supportive care alone [10]. This trial added an important piece of information because tamoxifen was found ineffective also in a HCC population with a high prevalence of alcohol-related liver cirrhosis. However, following a post-hoc unplanned subgroup analysis, the Authors suggested that tamoxifen might be effective in a population of patients without major hepatic insufficiency, i.e. those with Okuda stage I or II, and that new trials on tamoxifen are still warranted.

The aim of this paper is to externally validate the hypothesis of Barbare et al. [10] using the updated data of the CLIP-1 randomised trial [3].

## Methods

The methods of the CLIP-1 trial have been detailed elsewhere [1]. Brief information is given below.

CLIP-1 multicenter randomised trial had a pragmatic approach with large and simple inclusion criteria: all patients with a life expectancy longer than three months and diagnosed less than two years before were eligible for the study. Differently from other studies, this trial was not limited to patients with advanced disease, thus we had the opportunity to assess the possible efficacy of treatment with tamoxifen even in potentially curable subjects [3].

Patients were centrally randomised either to tamoxifen (40 mg/day) or supportive therapy using a minimization procedure with center, evidence of disease at entry and time from HCC diagnosis as stratification variables. Overall 496 subjects entered the study from 30 Italian institutions and 477 were evaluable for the analysis, 237 assigned to the tamoxifen arm, and 240 to the control arm.

Overall survival was the only endpoint of the trial and analyses were performed according to an intention-to-treat basis. Survival curves were drawn by the Kaplan-Meier method and compared by the logrank test.

CLIP score was estimated as reported before [11,12], using Child-Pugh stage, tumor morphology, alpha fetoprotein (AFP) and portal vein thrombosis as score components.

The hypothesis to be tested was that tamoxifen might be effective in patients with better prognosis, thus, according to the original study [10], two subgroups of the Okuda stage were derived (I/II and III/unknown). Similarly two subgroups of the CLIP score were obtained combining together the patients at better prognosis (scores 0 or 1) and at worse prognosis (scores equal to or greater than 2 or unknown).

## Results

Distributions of baseline variables by treatment arm are reported in Table 1, for Okuda subgroups, and Table 2, for CLIP subgroups.

Overall baseline variables were well balanced between the two treatment arms. As expected, more potentially curative treatments (surgery or percutaneous ethanol injection) were observed in the subgroup with the better prognosis. Less patients were classified in the better subgroup with the CLIP score than with the Okuda score.

**Figure 1:**
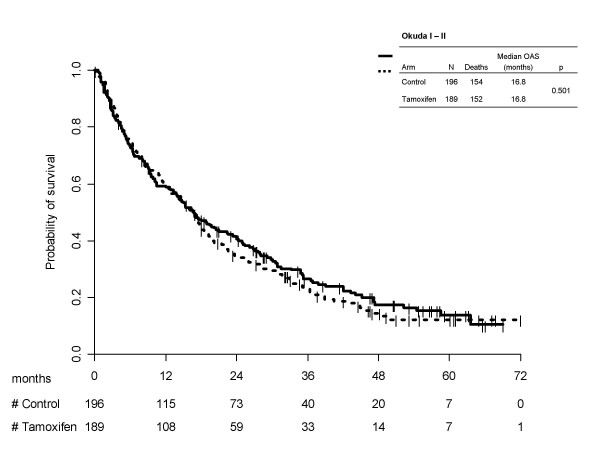
Kaplan-Meier estimated survival curves by treatment arm, according to Okuda subgroups I/II.

Kaplan-Meier survival curves for treatment arms, separately by Okuda subgroups are reported in Figure 1 (score I/II) and 2 (score III/unknown). In both cases tamoxifen was not effective (p = 0.501 and p = 0.650, respectively). In the first subgroup median survival times was equal to 16.8 (95%CI 12.7–18.5) months for tamoxifen and 16.8 (95%CI 13.5–22.4) months for the control arms; 1-year survival probabilities were equal to 58.8% (95%CI 51.7–65.8) and 59.4 (95%CI 52.5–66.2), respectively. Of course, in patients with Okuda stage III or unknown, prognosis was much worse, median survival times being equal to 8.9 (95%CI 5.5–14.5) months for tamoxifen and 13.1 (95%CI 6.0–28.0) months for control; 1-year survival probabilities were equal to 42.6% (95%CI 28.4–56.7) with tamoxifen and 50.8% (95%CI 35.7–65.8) without it.

Kaplan-Meier survival curves for treatment arms, separately by CLIP subgroups are reported in Figure 3 (score 0/1) and 4 (score ≥2/unknown). Once again no statistically significant differences were found between the two treatment arms (p = 0.734) in the subgroup at better prognosis and (p = 0.286) in the subgroup at worse prognosis). In the first subgroup median survival times were equal to 29.2 (95% CI 20.1–36.4) months with tamoxifen and 29.0 (95% CI 23.3–35.2) months without it; 1-year survival probabilities were equal to 80.9% (95% CI 72.5–89.3) with tamoxifen and 77.1% (95% CI 68.6–85.7) for the control arm. Prognosis was worse in patients with CLIP score >1 or unknown, median survival times being equal to 8.4 (95% CI 6.2–11.9) months for tamoxifen and 9.1 (95% CI 6.6–13.8) months for control; 1-year survival probabilities were equal to 41.1% (95% CI 33.2–49.0) with tamoxifen and 45.5% (95%CI 37.4–53.6) without.

## Discussion

In this paper we report a failed attempt to validate the Barbare's hypothesis [10] that tamoxifen might be effective in patients with better prognosis and confirm the previous evidence that there is no survival advantage for treating HCC patients with tamoxifen in addition to supportive therapy.

Because of the broad eligibility criteria applied in the CLIP-1 trial, we had already tested possible survival differences within subgroups defined by locoregional treatment [3]. Because of the strict correlation between prognosis and locoregional treatment, the present lack of significance was expected.

**Figure 2:**
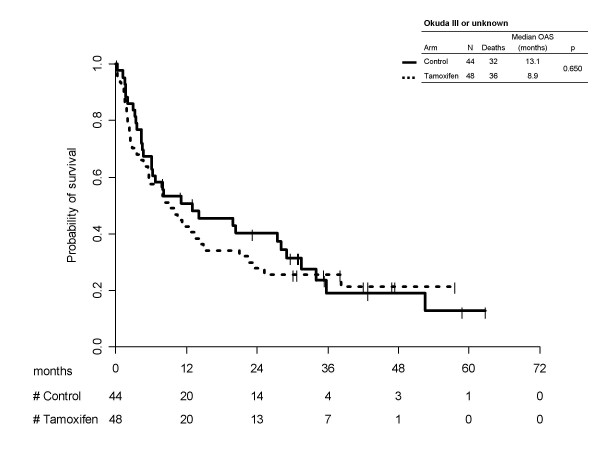
Kaplan-Meier estimated survival curves by treatment arm, according to Okuda subgroups III/unknown.

**Figure 3:**
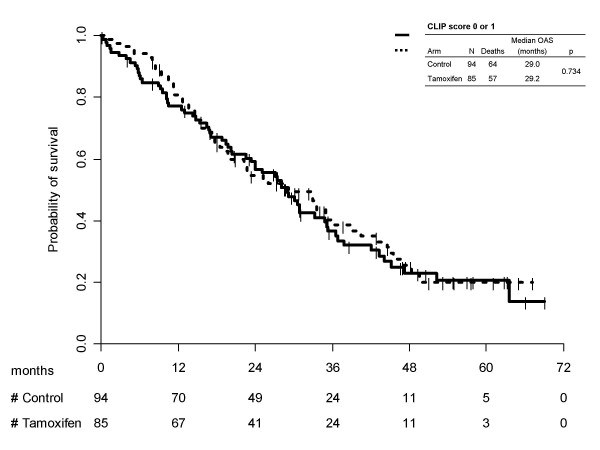
Kaplan-Meier estimated survival curves by treatment arm, according to CLIP subgroups 0/1.

We also tested the same hypothesis in subgroups defined according to the CLIP score, that currently is the most widely accepted and validated prognostic score for HCC [11,12]. CLIP scores 0 and 1 definitely identified patients without major hepatic insufficiency better than Okuda score (Figures 1a and 2a), but once again no evidence of tamoxifen effectiveness was found.

Our results substantiate the criticism against subgroup analyses, even more urgently when they are not previously planned. In multiple subgroup analyses both more false negative results, due to the smaller size of the groups, and more false positive results, due to multiple comparisons, are expected. Exploring data in search for differences possibly depending on certain baseline characteristics may be acceptable on its own, but subgroup findings must not be over-interpreted [13]. Although the power is usually limited, only statistical tests of interaction should be performed rather than subgroup-specific tests. Anyway, the interpretation strictly depends on whether findings are biologically plausible, how many analyses are done, whether they are planned ahead and how much strong is the statistical evidence, still being aware that most subgroup claims tend to exaggerate the truth [13,14].

Often, it is enough to correct for multiple testing so that results are no more statistically significant and conclusions not sounded; as would have been the case in the trial recently reported by Barbare et al [10].

Although Barbare et al. discussed their findings must be regarded with great caution, their conclusion was that new trials are warranted in the specific population without major hepatic insufficiency. That conclusion brought about this report, but we failed to substantiate it.

## Conclusion

In conclusion, we believe that the story of tamoxifen in HCC was definitively written; to date, neither further trials are warranted with this drug in HCC nor any use in clinical practice should be considered because of its clear lack of efficacy.

## Competing interests

The author(s) declare that they have no competing interests.

## Authors' contributions

CG and FP projected the study and drafted the manuscript;

EDM, MDM, BD and SP participated in acquisition of data and gave substantive intellectual contribution when revising it critically;

GS and AA performed the analysis of the data.

All authors read and approved the final manuscript.

## Pre-publication history

The pre-publication history for this paper can be accessed here:



## Figures and Tables

**Figure 4:**
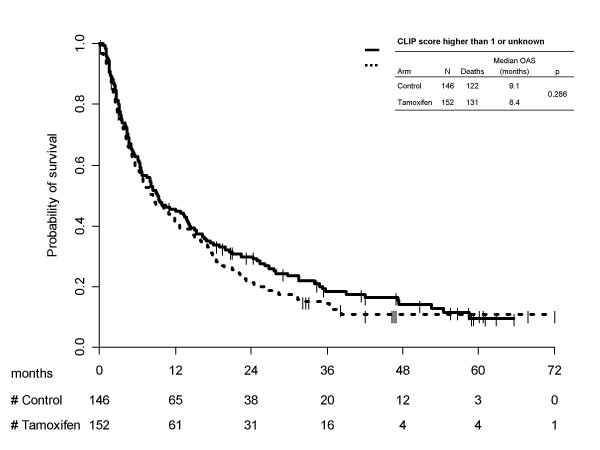
Kaplan-Meier estimated survival curves by treatment arm, according to CLIP subgroups >1/unknown.

**Table 1:** Distributions of baseline variables by treatment arm, according to prognostic Okuda subgroups

***Okuda category***	**I-II**		**III or unknown**	
	**Tamoxifen (n = 189)**	**Control (n = 196)**	**Tamoxifen (n = 48)**	**Control (n = 44)**
Median (range) age, *years*	66 (39–91)	67 (42–84)	62 (37–86)	66 (31–80)
Men	133 (70.4)	144 (73.5)	36 (75.0)	39 (88.6)
Evidence of disease at entry	178 (94.2)	184 (93.9)	40 (83.3)	39 (88.6)
Interval between diagnosis and entry				
0–6 months	150 (79.4)	161 (82.1)	44 (91.7)	38 (86.7)
7–12 months	20 (10.6)	15 (7.7)	1 (2.1)	4 (9.1)
13–24 months	19 (10.1)	20 (10.2)	6 (6.3)	2 (4.6)
Child-Pugh category				
A	90 (47.6)	98 (50.0)	25 (52.1)	20 (45.6)
B	82 (43.4)	75 (38.3)	13 (27.1)	11 (25.0)
C	17 (9.0)	23 (11.7)	9 (18.8)	12 (27.3)
unknown	0 (0.0)	0 (0.0)	1 (2.1)	1 (2.3)
Locoregional treatment				
Surgery	11 (5.8)	12 (6.1)	7 (14.6)	3 (6.8)
Percutaneous Ethanol Injection	61 (32.3)	72 (36.7)	3 (6.3)	6 (13.6)
Transarterial chemoembolization	31 (16.4)	33 (16.8)	11 (22.9)	5 (11.4)
none	86 (45.5)	79 (40.3)	27 (56.3)	30 (68.2)

**Table 2:** Distributions of baseline variables by treatment arm, according to prognostic CLIP subgroups

***Clip category***	***0–1***		***2–6 or unknown***	
	**Tamoxifen (n = 85)**	**Control (n = 94)**	**Tamoxifen (n = 152)**	**Control (n = 146)**
Median (range) age (years)	66 (37–91)	66 (31–80)	65 (39–86)	67 (42–84)
Men	57 (67.1)	68 (72.3)	112 (73.7)	115 (78.8)
Evidence of disease at entry	77 (90.6)	86 (91.5)	141 (92.8)	137 (93.8)
Interval between diagnosis and entry				
0–6 months	85 (100.0)	94 (100.0)	109 (71.7)	105 (71.9)
7–12 months	0 (0.0)	0 (0.0)	21 (13.8)	19 (13.0)
13–24 months	0 (0.0)	0 (0.0)	22 (14.5)	22 (15.1)
Child-Pugh category				
A	62 (72.9)	67 (71.3)	53 (34.9)	51 (34.9)
B	23 (27.1)	27 (28.7)	72 (47.4)	59 (40.4)
C	0 (0.0)	0 (0.0)	26 (42.6)	35 (24.0)
unknown	0 (0.0)	0 (0.0)	1 (0.7)	1 (0.7)
Locoregional treatment				
Surgery	10 (11.8)	10 (10.6)	8 (5.3)	5 (3.4)
Percutaneous Ethanol Injection	34 (40.0)	46 (48.9)	30 (19.7)	32 (21.9)
Transarterial chemoembolization	18 (21.2)	17 (18.1)	24 (15.8)	21 (14.4)
none	23 (27.1)	21 (22.3)	90 (59.2)	88 (60.3)
